# In the Gut’s Microbial Community, One Plus One Equals Many (Effects)

**DOI:** 10.1371/journal.pbio.0040447

**Published:** 2006-11-28

**Authors:** Richard Robinson

The instructions for assembling an adult human being include adding ten parts microbial cells to one part human cells. The parts list on the microbial side have likely changed as our diets, lifestyles, and biosphere have undergone significant changes. Reports are appearing that show that our microbial partners’ genomes (the human microbiome) encode physiologic capacities that complement our own deficiencies. For example, the microbiome gives rise to a large assemblage of microbial enzymes not found in humans that break down and extract calories from food we ingest and inactivate potentially toxic compounds that we consume. This leads to a sobering thought: that technological advances aimed at reducing food spoilage and our contact with environmental microbes have instead compromised the services of our microbial partners and made us more vulnerable to certain maladies, including digestive disorders.

Against this background, a class of microbes, most derived from fermented dairy products, have been promoted as benefiting human health if consumed on a regular basis. They bear the moniker “probiotics” and may be able to restore features lost during the hypothesized sanitization of our 21st century microbiota. But a chorus of skeptical inquirers wonder how, and whether, probiotics work. Do they alter the properties of our incredibly complex community of gut microbes? Can a few billion influence many trillions? Do they communicate directly with their host, or do they require a microbial brigade to translate their effects? If so, what precisely are the effects on our biology, and do these effects occur in all individuals?

Justin Sonnenburg, Christina Chen, and Jeffrey Gordon address these questions in a new study by turning to a simplified model of the human gut. Their starting point was a collection of germ-free mice who had spent their entire lives in sterile plastic bubbles munching on microbe-free chow and water. Sonnenburg et al. then introduced a prominent member of the normal human gut community (the almost unpronounceable *Bacteroides thetaioatomicron*) with or without *Bifidobacterium longum*—a minor resident of the community and a probiotic. Using a custom “community” GeneChip to monitor gene expression in the two bacterial species, another GeneChip to monitor the gene expression in the host gut, plus mass spectrometry, they show how each organism affects the other as well as the mouse cells that line the gut.

**Figure:**
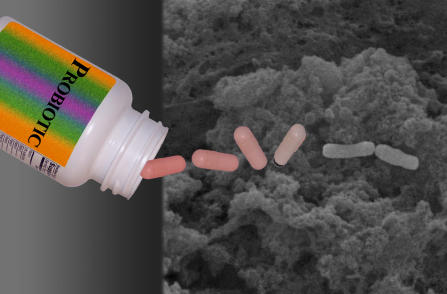
Probiotics are widely used preparations of live bacteria intended to improve human health. Methods are now in hand to begin to define their impact on the human gut microbiota and their host. (Image: Justin Sonnenburg, Jamie Dant, Laura Kyro, and Jeffrey Gordon)

*B. thetaiotaomicron* expands its capacity to consume a broad range of otherwise indigestible polysaccharides when it co-exists with *B. longum*; this involves preferential use of dietary plant glycans over host mucus glycans. *B. longum* does not expand its dining habits, but rather shifts away from one class of polysaccharides that its microbial neighbor is using (mannosides), and turns its attention toward another (xylosides). Similar to cohabitation with *B. longum*, another fermented dairy product–associated species, *Lactobacillus casei*, induces expansion of *B. thetaiotaomicron*’s polysaccharide digestive capabilities, but *B. longum*’s close relative *Bifidobacterium animalis* does not. These responses, documented in a highly defined and controlled system, offer a first glimpse at how microbes respond to one another in the gut and emphasize that not all probiotic species affect the microbiota in the same manner. The host acknowledges when *B. thetaiotaomicron* and *B. longum* are present together by mobilizing the expression of suites of genes, including those involved in modulating the activity of the immune system.

The approaches described in this study should be generally useful for defining ways that we and our modern microbiota may be affected by probiotics and for developing a knowledge base that can be applied to humans in well-controlled clinical trials. Ultimately, the goal is to develop rational ways to optimize the performance of our microbial partners so that our health as a “superorganism” can be improved. For more on probiotics, see the Primer in the December issue; DOI: 10.1371/journal.pbio.0040430.

